# Discovering Long COVID Symptom Patterns: Association Rule Mining and Sentiment Analysis in Social Media Tweets

**DOI:** 10.2196/37984

**Published:** 2022-09-07

**Authors:** Surani Matharaarachchi, Mike Domaratzki, Alan Katz, Saman Muthukumarana

**Affiliations:** 1 Department of Statistics University of Manitoba Winnipeg, MB Canada; 2 Department of Computer Science Western University London, ON Canada; 3 Departments of Community Health Sciences and Family Medicine University of Manitoba Winnipeg, MB Canada

**Keywords:** COVID-19, long COVID symptoms, social media analysis, association rule mining, bigram analysis, natural language processing, Twitter, content analysis, data mining, infodemiology, health information

## Abstract

**Background:**

The COVID-19 pandemic is a substantial public health crisis that negatively affects human health and well-being. As a result of being infected with the coronavirus, patients can experience long-term health effects called long COVID syndrome. Multiple symptoms characterize this syndrome, and it is crucial to identify these symptoms as they may negatively impact patients’ day-to-day lives. Breathlessness, fatigue, and brain fog are the 3 most common continuing and debilitating symptoms that patients with long COVID have reported, often months after the onset of COVID-19.

**Objective:**

This study aimed to understand the patterns and behavior of long COVID symptoms reported by patients on the Twitter social media platform, which is vital to improving our understanding of long COVID.

**Methods:**

Long COVID–related Twitter data were collected from May 1, 2020, to December 31, 2021. We used association rule mining techniques to identify frequent symptoms and establish relationships between symptoms among patients with long COVID in Twitter social media discussions. The highest confidence level–based detection was used to determine the most significant rules with 10% minimum confidence and 0.01% minimum support with a positive lift.

**Results:**

Among the 30,327 tweets included in our study, the most frequent symptoms were brain fog (n=7812, 25.8%), fatigue (n=5284, 17.4%), breathing/lung issues (n=4750, 15.7%), heart issues (n=2900, 9.6%), flu symptoms (n=2824, 9.3%), depression (n=2256, 7.4%) and general pains (n=1786, 5.9%). Loss of smell and taste, cold, cough, chest pain, fever, headache, and arm pain emerged in 1.6% (n=474) to 5.3% (n=1616) of patients with long COVID. Furthermore, the highest confidence level–based detection successfully demonstrates the potential of association analysis and the Apriori algorithm to establish patterns to explore 57 meaningful relationship rules among long COVID symptoms. The strongest relationship revealed that patients with lung/breathing problems and loss of taste are likely to have a loss of smell with 77% confidence.

**Conclusions:**

There are very active social media discussions that could support the growing understanding of COVID-19 and its long-term impact. These discussions enable a potential field of research to analyze the behavior of long COVID syndrome. Exploratory data analysis using natural language processing methods revealed the symptoms and medical conditions related to long COVID discussions on the Twitter social media platform. Using Apriori algorithm–based association rules, we determined interesting and meaningful relationships between symptoms.

## Introduction

COVID-19, a transmissible disease caused by the SARS-CoV-2 virus, has become a substantial public health crisis that negatively affects people’s health and well-being. Most people with COVID-19 recover entirely within weeks. However, some people still experience symptoms after their initial recovery, even those who had mild symptoms with their initial infection. Others develop new symptoms related to their COVID-19 illness. These people sometimes describe themselves as “long haulers” [1]. This syndrome has been described as post–COVID-19 or “long COVID-19” [2]. It is crucial to identify these symptoms as they may negatively impact the day-to-day lives of those with this affliction for a substantially long period. Breathlessness, fatigue, and brain fog are the 3 most common symptoms that patients with long COVID have consistently reported, often months after the onset of the COVID-19 disease [3,4].

Social media has become a substantial part of our lives. People use it to connect with others and share their thoughts, emotions, and experiences about any current topic, often without revealing their identity [5]. Hence, social media platforms such as Twitter, Facebook, and Instagram have had a massive impact on society and gained considerable research attention [6]. There are extensive chains of discussions on these platforms about the long COVID syndrome (LCS). Thus, analyzing social media conversations of long COVID–related patients from various sources provides an opportunity to understand the relationship between symptoms and their consequences.

According to the World Health Organization clinical case definition [4], “long COVID-19 condition occurs in individuals with a history of probable or confirmed SARS CoV2 infection, usually three months from the onset of COVID-19, with symptoms that last for at least two months.” Health-related organizations, such as the Mayo Clinic [7], National Health Service [8], Centers for Disease Control and Prevention [9], and World Health Organization [4], have identified different lists of symptoms related to long COVID, and a summary is presented below. The results of recent research that has been conducted using self-reported long COVID symptoms on Twitter are presented [10]. These symptoms were identified by manually reading 165 tweets from July 20, 2020, to July 29, 2020.

There are, however, very active ongoing social media discussions that could support the growing understanding of the illness and its long-term impact. These discussions provide the opportunity to access publicly available data from multiple individuals on Twitter to analyze long COVID symptoms. However, manually discovering the knowledge in a large volume of unstructured texts is increasingly problematic. Hence, automated natural language processing (NLP) methods have been introduced to do this task effectively and accurately [11,12]. The importance of using NLP methods in health sciences has been increasingly recognized over recent years [13]. Even though previous research has identified a list of symptoms, extracting and grouping the symptoms discussed in tweets into categories makes them easier to analyze and find the relationships between the most common symptoms. Manually handling this task is challenging; thus, NLP tools represent an opportunity to extract hidden information from unstructured Twitter text data.

Association rules are considered a useful tool as they offer the possibility to conduct intelligent diagnoses, extract invaluable information, and build important knowledge quickly and automatically while identifying relationships within and between variables [14]. Thus, we used association rule mining (ARM) to discover relationships among long COVID symptoms based on the symptoms revealed from text data [15]. The mining process of association rules requires setting a minimum confidence and support threshold to describe association rules that are meaningful. Therefore, we identified association rules based on a minimum confidence threshold of 10% and a minimum support threshold of 0.1%.

This study sought to achieve the 2 goals. The first goal was to identify the symptoms and medical conditions related to long COVID that were discussed on the Twitter social media platform. The second goal was to determine the patterns of symptoms and their associations. By accomplishing these objectives, this work will ultimately help physicians identify the behavior of the patients with long COIVID. This paper provides new ideas for symptom mining and reveals the internal relationship between symptoms and their application value. Thus, the work has theoretical and practical implications.

## Methods

### Data Collection

We collected worldwide, long COVID–related, and English-language tweets between May 1, 2020, and December 31, 2021, to create our data set of about 1 million tweets. We used the *Snscrape* module in Python (version 3.8; Python Software Foundation) [16] to scrape the web-based tweet text from tweets that match the keyword “LongCovid.” Streaming English-language tweets from multilingual tweets is computationally intensive, because most nonnative English-speaking countries use their native languages rather than English to express their feelings on social media; thus, it requires translating each tweet to English if we wish to analyze them. Therefore, we limited the data set to English-language tweets. The most useful attributes of our data set were ID (Number), time created (DateTime), original text (Text), and language (Text), which we used to filter English-language tweets only. We removed duplicates of tweet text from our data set. This processing necessarily removed any retweets in our data set, which is consistent with our goals of collecting a data set that represents patient experiences. The average tweet length of the initial data set was 32.56 words.

We reduced the data set to 127,848 tweets by limiting the population to those with COVID-19. To do this, we refined the tweets to ensure that all the tweets reflect personal experiences with long COVID. We first considered tweets containing the pronoun “I” and the word “covid” as we wanted to extract tweets from people with COVID-19 or long COVID. Subsequently, we removed tweets containing words that explain users’ opinions, as many people discuss long COVID without necessarily having COVID-19. The set of the words or phrases we considered is listed in Table 1 with the percentage of tweets including the specific word. The phrase “I feel” may include tweets that express the experience of symptoms; however, to remove the possibility of opinion, we removed these tweets. We also observed a number of tweets (3394/148,672, 2.28%) that talked about similar conditions, especially chronic fatigue syndrome. To eliminate the context related to chronic fatigue syndrome, we removed tweets containing the keyword “cfs.” Our data set was further reduced to identify only the patients discussing their experiences with the symptoms. First, we created a list of long COVID symptoms discussed in different literature sources, as shown in Table 2. We then found the most common symptoms by comparing them with the preprocessed word corpus shown in Table 3.

**Table 1:** The list of words that explain users’ opinions rather than their experience.

Word or phrase	Tweets (N=148,672), n (%)
“opinion”	641 (0.43)
“I believe”	1194 (0.8)
“I think”	6861 (4.61)
“I feel”	2006 (1.35)
“may be” OR “maybe” OR “might”	7582 (5.1)
“perhaps”	750 (0.5)

**Table 2:** List of long COVID symptoms identified by different sources in the literature.

	Symptom	Mayo Clinic	NHS^a^	CDC^b^	WHO^c^	Singh and Reddy [10]
1	Extreme tiredness (fatigue)	✓	✓	✓	✓	✓
2	Shortness of breath or difficulty breathing	✓	✓	✓	✓	✓
3	Cough	✓	✓	✓	✓	✓
4	Joint pain	✓	✓	✓	✓	
5	Chest pain or tightness	✓	✓	✓	✓	✓
6	Problems with memory and concentration (“brain fog”)	✓	✓	✓	✓	✓
7	Difficulty sleeping (insomnia)		✓	✓	✓	✓
8	Muscle pain	✓		✓	✓	✓
9	Headache	✓	✓	✓	✓	✓
10	Fast or pounding heartbeat (heart palpitations) or tachycardia	✓	✓	✓	✓	✓
11	Loss of smell	✓	✓	✓	✓	✓
12	Loss of taste	✓	✓	✓	✓	✓
13	Depression or anxiety					
14	Fever	✓	✓		✓	
15	Dizziness (light-headedness)	✓	✓	✓	✓	✓
16	Worsened symptoms after physical or mental activities	✓		✓		
17	Pins-and-needles feeling		✓	✓	✓	
18	Tinnitus and earaches		✓		✓	✓
19	Diarrhea		✓	✓	✓	
20	Stomach aches		✓	✓		
21	Loss of appetite		✓			✓
22	Sore throat		✓			
23	Rash			✓		
24	Mood changes			✓		
25	Changes in menstrual period cycles			✓	✓	
26	Abdominal pain				✓	✓
27	Neuralgias				✓	
28	Allergies				✓	
29	Body pain					✓
30	Nausea					✓
31	Weakness					✓
32	Numbness					✓

^a^NHS: National Health Service.

^b^CDC: Centers for Disease Control and Prevention.

^c^WHO: World Health Organization.

**Table 3:** Preprocessed word corpus of stemmed symptoms.

Group	Symptoms
Brain fog	“brain fog,” “brain,” “fog,” “memori,” “mental,” “rememb,” “concentr,” “mind,” “remind,” and “focus”
Fatigue	“fatigu,” “tire,” and “exhaust”
Lung	“lung,” “breathless,” and “breath”
Cannot walk	“cant walk,” “struggl walk,” “unabl walk,” “couldnt walk,” “bare walk,” “unaid walk,” and “stair walk”
Depress	“depress,” “mood,” “stress,” and “anxieti”
Lose weight	“lose weight” and “loss weight”
Insomnia	“cant sleep” and “insomnia”
Diarrhea	“diarrhea” and “diarrhoea”
Dizziness	“dizz” and “lighthead”
Heart	“heart,” “heart palpit,” “tachycardia,” “dysautonomia,” and “arrhythmia”
Others	“headach,” “neck,” “arm,” “muscl pain,” “cough,” “chest pain,” “flu,” “joint pain,” “pain,” “rash,” “fever,” “loss smell,” “loss tast,” “cold,” “earach,” “vomit,” “chill,” “nausea,” “faint,” “gain weight,” “trauma,” “bodi,” “bleed,” “appetit,” “sore throat,” “pin needl,” “numb,” “tinnitu,” “buzz,” “hairfall,” “nose,” “stomach,” “menstrual,” and “abdomin”

### Data Preprocessing

Data preprocessing was mainly used to clean the raw data by following specific steps to achieve better results for further evaluations. We preprocessed our initial data to ensure quality by developing a user-defined preprocessing function based on *Natural Language Toolkit* (*NLTK*; a Python library for NLP) [15].

**Figure 1:**
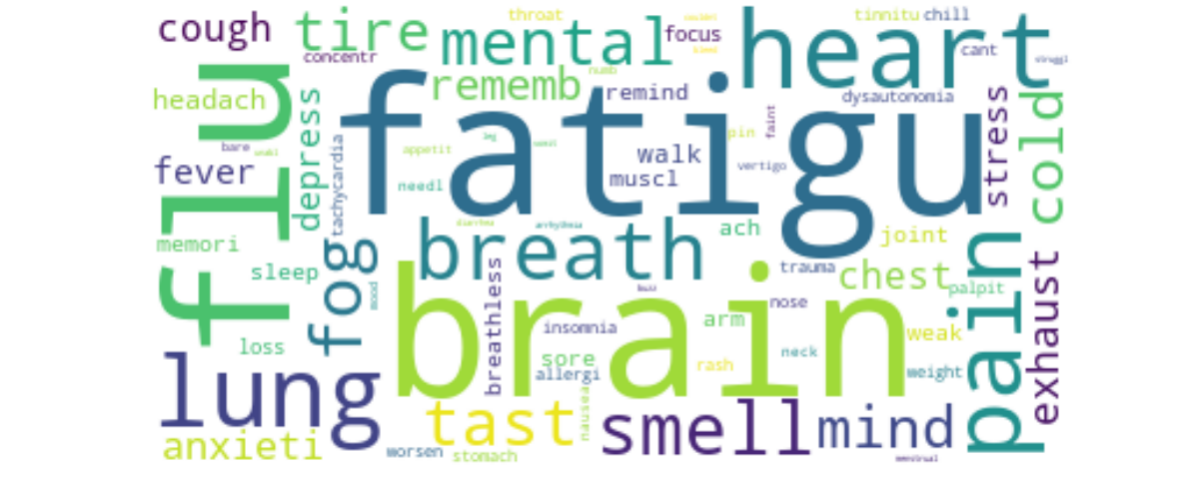
Word cloud of the list of 73 words after stemming.

The preprocessing plan was as follows. First, we removed the hashtag symbol and its content (eg, COVID-19, @users, and URLs) from the texts because the hashtag symbols and the URLs did not contribute to the text analysis. We also removed all non-English characters (non–American Standard Code for Information Interchange characters) because the study focused on analyzing tweets in English. We then removed repeated words and stop words identified by *NLTK*. Special characters, punctuation, and numbers from the data set were also removed as they did not help the detection of comments with profanity. We conducted a sentiment analysis to gauge how patients feel about long COVID–related topics, and the results are discussed in the next section. The primary focus of the study was symptom mining; therefore, we created a data set for patients with symptom information using a predefined set of keywords. Figure 1 shows the word cloud of the set of keywords accounted for initial tweets extraction.

**Figure 2:**
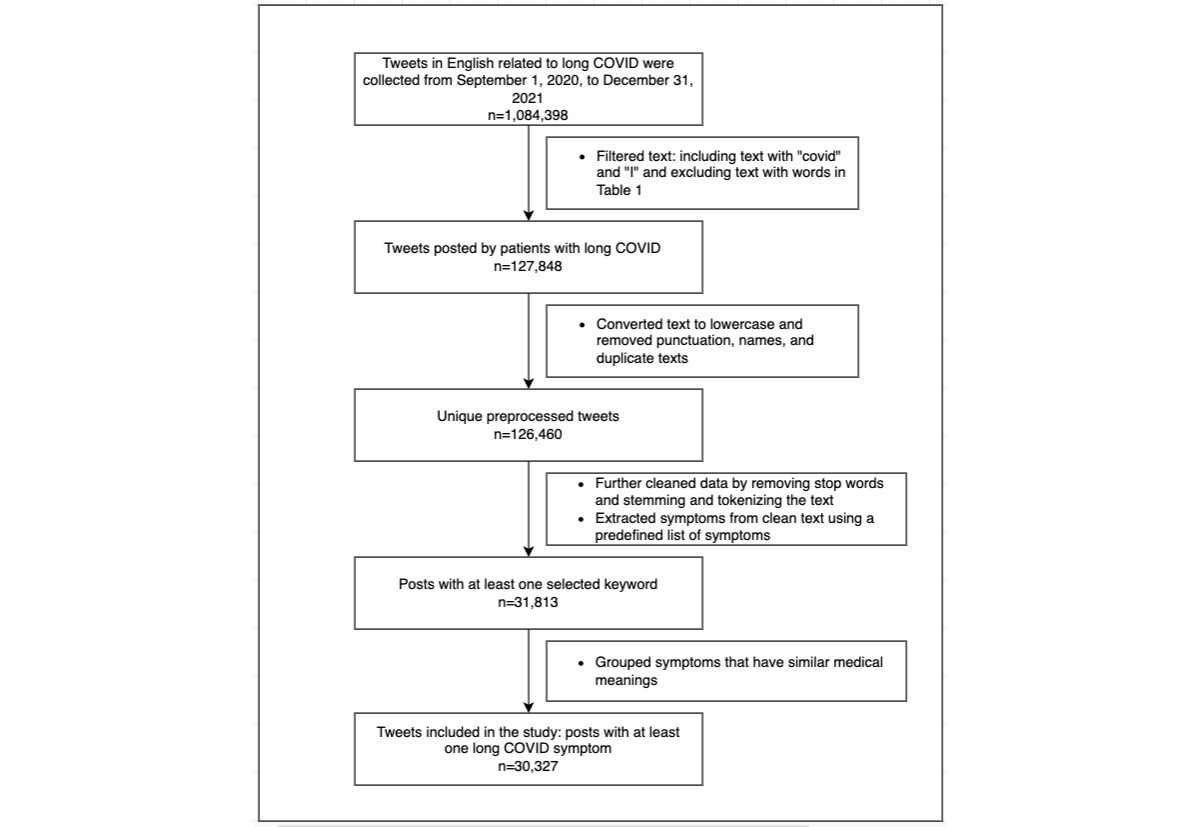
Data collection and preprocessing process.

**Figure 3:**
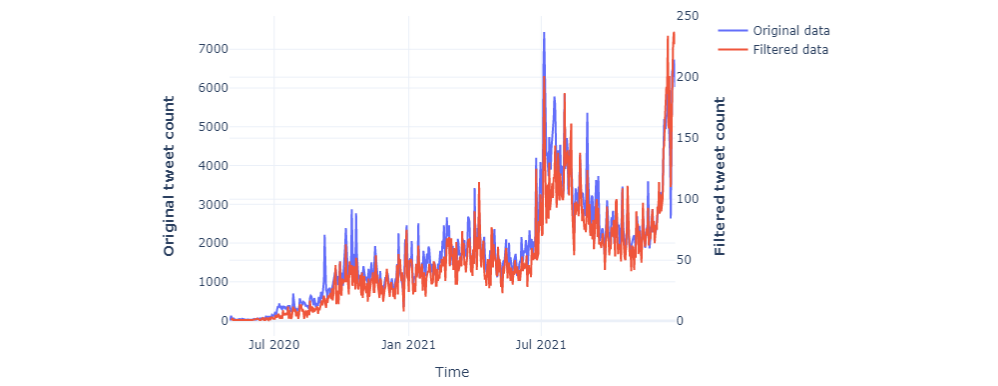
Time series plot for originally obtained data and the data considered for the study.

Stemming reduced inflected words to their word stem, base, or root form, whereas tokenization was used to split each sentence into smaller parts of a word. Figure 2 shows how the data collection, cleaning, and preprocessing steps were conducted and how the final data set with 30,327 tweets was obtained for analysis in the study. After performing cleaning and preprocessing steps, the average tweet length was reduced from 32.56 to 19.07 words. Initially obtained data (original data) and the data considered for the study (filtered data) have been plotted in the same time series plot (Figure 3). The filtered data represented the original data appropriately.

### Sentiment Analysis

To measure the sentiment expressed via Twitter on long COVID, we used sentiment analysis, a specific type of NLP, computational linguistics, and text analysis [16,17]. The subjective information from tweets was analyzed and extracted to classify the text into 3 classes, namely positive, negative, and neutral. If the polarity was >0, the text was categorized as “positive,” and if the polarity was <0, the text was categorized as “negative.” Texts with 0 polarity values were classified as “neutral.” The pie charts in Table 4 show the categorization of the sentiment of each text related to long COVID in 2 stages—for all the posts and posts with at least one long COVID symptoms, respectively. The positive sentiment was reduced from 53.1% (67,153/126,460) to 49.8% (15,102/30,327) when we only considered the tweets with symptoms.

**Table 4:** Classification of the sentiment scores.

	Classes		
	Positive	Negative	Neutral
Sentiment scores of all posts, %	53.1	45.2	1.66
Sentiment scores of posts with at least one long COVID symptom, %	49.8	48.7	1.53

At this stage, we calculated the sentiment polarity of each cleaned and preprocessed tweet using the *TextBlob* library. *TextBlob* is a Python library that supports complex analysis and operations on textual data. It is built on the *NLTK* library [15] and provides a simple API for performing several NLP tasks, such as sentiment analysis, part-of-speech tagging, noun phrase extraction, classification, and translation.

### Collocations

We also understand that symptoms often appear as more than 1 word in texts. Therefore, finding meaningful symptoms with only 2 words was a particular task in this study. Many valuable text analyses are based on the relationships between words, examining which words tend to follow others immediately or co-occur. Therefore, we analyzed the relationship between 2 words of each bigram in tweets and identified which long COVID symptoms appear as a combination of words. We used the *NLTK* library in Python [15] to identify biwords from the texts and filtered them using the preprepared list of symptoms to only get biwords that can be meaningful symptoms of long COVID when appearing together.

To identify the meaningful biwords, we used the collocation feature in words that reveal a phrase consisting of more than 1 word. Still, these words more commonly co-occur in a given context than their individual word parts. We used several bigram-association measures [18] to filter out the most meaningful collocations.

1. Pointwise mutual information (PMI): the PMI score for 2 words, *w*^1^ and *w*^2^, is as follows:



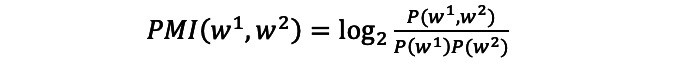



The main intuition is that it measures how much more likely the words are to co-occur than to occur independently. However, the main disadvantage of this method is that it is very sensitive to a rare combination of words. We handled this issue by using the frequency filter of words.

2. Two-tailed *t* test with a frequency filter: When conducting a *t* test, we can consider testing the below hypothesis with a 5% level of significance.

*H*_0_: Words (*w*^1^*, w*^2^) occur with probability *µ*: 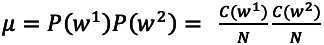


versus

*H*_1_: Words (*w*^1^*, w*^2^) do not occur with probability *µ*.

Where *C* represents the count of each word and *N* is the total number of words in the corpus.

The test statistic is as follows:



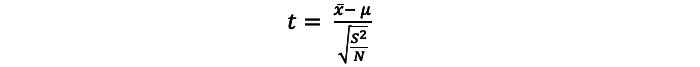



where, 
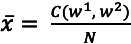
 for Bernoulli trial: 



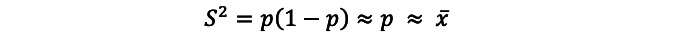



We also avoided the sensitivity to rare cases by filtering the biwords according to the frequencies. Normality assumption is one of the disadvantages of using the *t* test.

3. Chi-square test: The null hypothesis of the chi-square test assumes that words (*w*^1^, *w*^2^) are independent, just like in the *t* test. The chi-square test statistic is computed as follows:



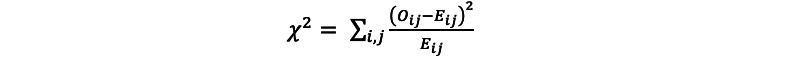



**Figure 4:**
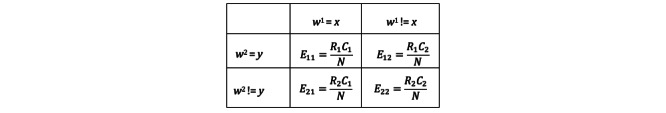
Bigram contingency table for a bigram (*x y*): estimated frequencies (*E_ij_*). *R_i_* and *C_j_* are the row and column totals, respectively. *N* indicates the total number of biwords in the texts.

The observed frequencies (*O_ij_*) and expected frequencies (*E_ij_*) can be calculated using the bigram contingency tables presented in Table 5 and Figure 4.

**Table 5:** Bigram contingency table for a bigram (*x y*): observed frequencies (*O_ij_*). z_*_ indicates words that are not *x* nor *y*. *C* (*w_i_ w_j_*) represents the biword count where *w_i_* and *w_j_* appear together. *R_i_* and *C_j_* are the row and column totals, respectively. *N* indicates the total number of biwords in the texts.

	*w*^1^ = *x*	*w*^1^ != *x*	
*w*^2^ = *y*	*O*_11_ = *C* (*x y*)	*O*_2__1_ = *C* (*z* *y*)	*R*_1_
*w*^2^ != *y*	*O*_21_ = *C* (*x z*)	*O*_22_ = *C* (*z*_1_ *z*_2_)	*R*_2_
	*C*_1_	*C*_2_	*N*

### Association Rule Mining

In many applications, implications between different situations naturally arise. We refer to these implications as associations. These associations can be discovered and quantified using relational knowledge. “Relational knowledge identifies how concepts/entities are related and how concepts and their relations are defined or described by models” [19]. For example, in the Twitter symptom analysis, some symptoms rules can be determined, such as “2.7% of patients with long COVID with loss of taste also experience loss of smell” or “patients with lung/breathing problems and loss of taste are likely to experience loss of smell with 77% confidence.” These rules are called association rules, and the correlation analysis is known as association mining.

ARM [20] has become an active research field in the data mining community that can solve various problems in health care. Recently, different incremental algorithms have been proposed for mining association rules to discover hidden relationships between symptoms and diseases. In this analysis, ARM was used to obtain insights into the poorly understood LCS by demonstrating the relationships and patterns among the symptoms described in the tweets. We addressed the problem of automatically identifying new and useful symptom patterns in the data of patients with long COVID using an Apriori rule–based data mining algorithm [16]. Recently, other incremental rule-based algorithms, such as Eclat [21], McEclat [22], Direct Hashing and Pruning [23], AprioriTID [24], and MsApriori [25], have also been introduced for mining association rules to discover hidden relationships between item sets and enhance the collection of rare frequent events. We used the Apriori algorithm in this paper as it is a well-understood and well-used algorithm out of this category. Computations and related experiments were done using Python.

We can help define LCS for future research and patient care by describing these relationships among symptoms. These patterns expose the combination of the symptoms that co-occur, as it is helpful to know how 1 symptom or set of symptoms is associated with others. An association rule between a set of symptoms X and a set of symptoms Y is expressed in the form X → Y. It is interpreted as “patients with symptoms X are likely to have symptoms Y.” Generally, the effectiveness of discovered rules is measured in terms of support, confidence, and lift.

1. Support: Support indicates how frequently the item set appears in the data set.



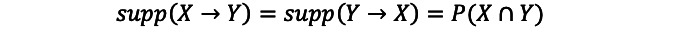



2. Confidence: Confidence is the percentage of all transactions satisfying X that also satisfy Y.



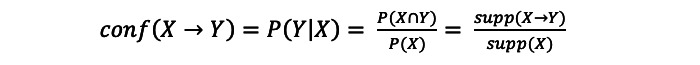



3. Lift: If the lift is >1, that lets us know the degree to which 2 occurrences are dependent on one another and makes these rules potentially helpful in predicting the consequences in future data sets.



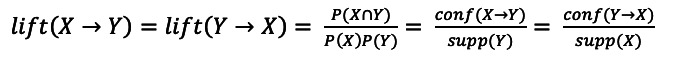



Thus, based on the analysis of long COVID symptoms, we can mine the association rules among symptoms and quantify their characteristics, such as confidence, support, and lift.

## Results

### Symptoms and Medical Conditions Related to Long COVID That Were Discussed on Twitter

Information was extracted for a total of 1,084,398 individual tweets, of which 34,022 had reported long COVID symptoms (Figure 1). The first 5 results obtained from each collocation method discussed in the previous section are shown in Table 6. After observing the results, we determined that the *t* test with a filter method achieves acceptable results. We also had to manually select the meaningful biwords with a test statistic >5 and that have a unique meaning only when they are together. We identified 20 such stemmed biwords that appeared together frequently, namely “brain fog,” “loss taste,” “loss smell,” “chest pain,” “cant walk,” “barely walk,” “unable walk,” “struggle walk,” “couldnt walk,” “stair walk,” “joint pain,” “muscle pain,” “leg pain,” “lose weight,” “loss weight,” “gain weight,” “cant sleep,” “sore throat,” “pin & needle,” and “heart palpitation.”

**Table 6:** First 5 biwords selected from each method.

Rank	Pointwise mutual information	*t* test with filter	Chi-square test
1	(runni, nose)	(brain, fog)	(brain, fog)
2	(pin, needl)	(mental, health)	(glandular, fever)
3	(shortterm, memori)	(chronic, fatigue)	(runni, nose)
4	(glandular, fever)	(tast, smell)	(mental, health)
5	(hay, fever)	(viral, fatigue)	(sore, throat)

**Figure 5:**
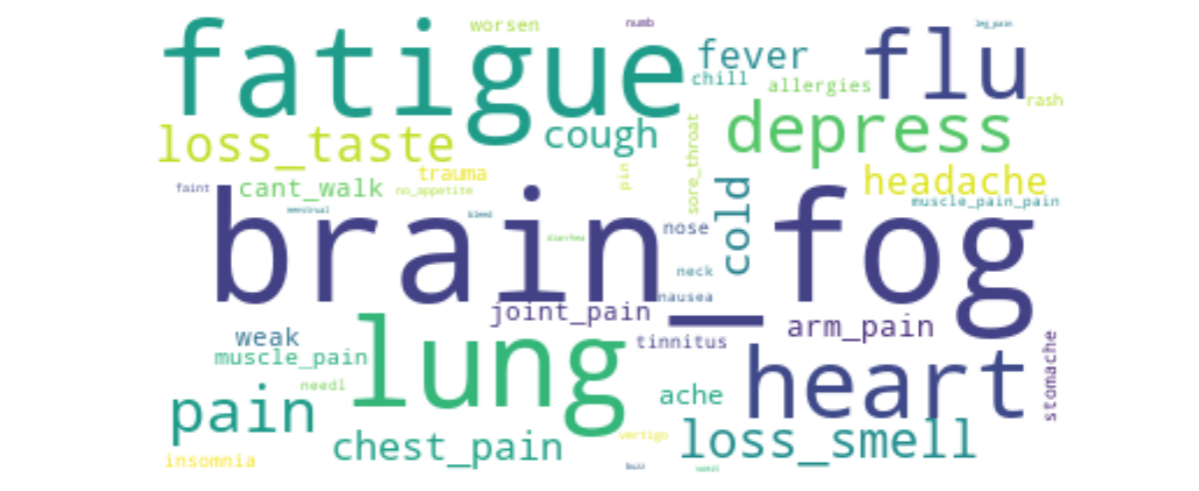
Word cloud of the list of 44 identified symptoms.

**Figure 6:**
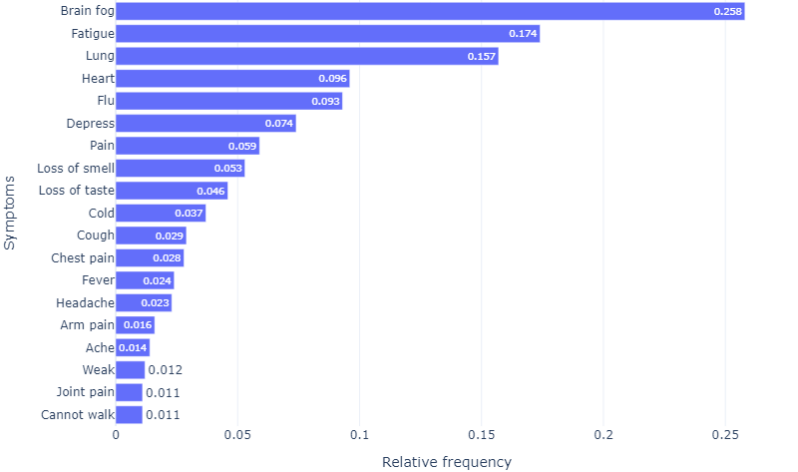
Relative frequency of symptoms in patients with long COVID.

Since some biwords have a similar medical meaning, we grouped similar words into categories for analysis (Table 3). After grouping symptoms, the total number of symptoms to be analyzed was decreased from 73 to 44. Figure 5 shows the reduced set of symptoms, and the size parameter of the word cloud indicates the frequency of each symptom appearing in the tweets. Among the 30,327 tweets, brain fog (n=7812, 25.8%) was the most common symptom, followed by fatigue (n=5284, 17.4%), breathing/lung issues (n=4750, 15.7%), heart issues (n=2900, 9.6%), flu symptoms (n=2824, 9.3%), depression (n=2256, 7.4%), and pain where the site is not explicitly mentioned (n=1786, 5.9%). Loss of smell and taste, cold, cough, chest pain, fever, headache, and arm pain were each reported in 1.6% (n=474; arm pain) to 5.3% (n=1616; loss of smell) of the tweets. Symptoms such as ache, weakness, joint pain, walking difficulties, muscle pain, trauma, allergies, worsened symptoms, tinnitus, insomnia, nose-related issues, stomach, and chills were reported in 1% of the tweets, whereas the rest of the symptoms were in less <1% of the tweets (Figure 6).

**Figure 7:**
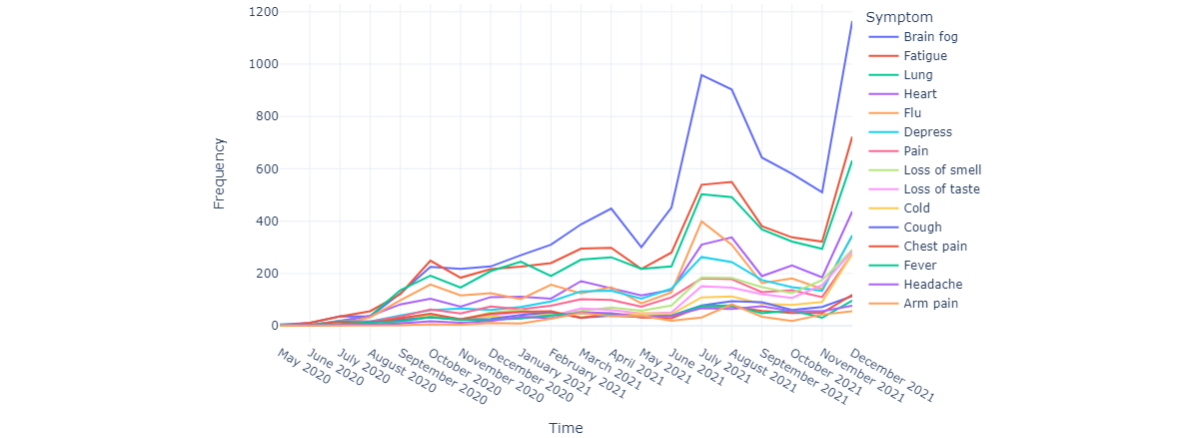
The 15 most frequent long COVID symptoms by time.

Figure 7 presents how each symptom appears in discussions over time, and the pattern is similar to the number of tweets shown in Figure 3. Hence, there is no noticeable difference in symptoms with time.

### Symptom Rules

Our study considered each tweet as a single transaction coming from a single individual. We applied the ARM algorithm to the symptom data considering 1 tweet as 1 transaction and identified symptom rules. The ARM algorithm using symptom transactions aimed to construct frequent item sets, having at least a user-specified threshold. Thus, we set a “confidence” threshold of 0.1 or 10%. We set up a minimum support threshold value above 0.001 and a “lift” greater than 1 for positively correlated rules. We discovered 57 significant rules for the data that included symptom-only information and presented them in Table 7.

**Figure 8:**
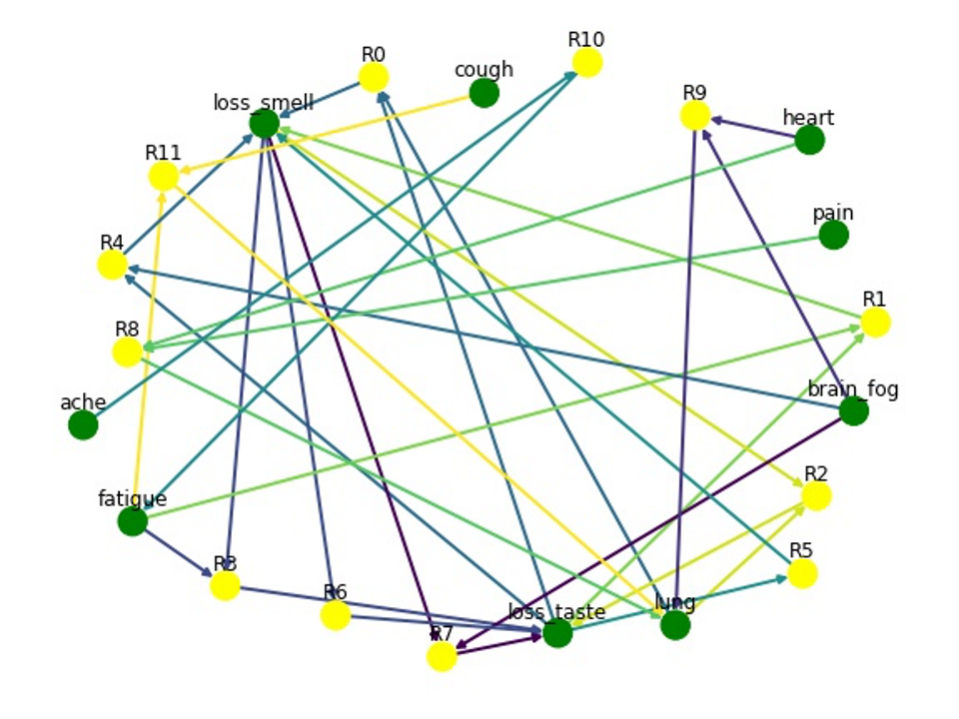
Association rules visualization. R: rule.

The highest confidence level–based detection was used to determine the most significant rules. Among the top 12 rules that have confidence >0.3, loss of smell and loss of taste were the most common consequent symptoms, followed by lung/breathing problems and fatigue. If a patient had lung/breathing problems and loss of taste, there was a 77% confidence that they had loss of smell. Similarly, patients with fatigue and loss of taste also had loss of smell as a consequent symptom. The top 12 rules are visualized in Figure 8. The following is a description of rule number 11, represented by R11 in a yellow node. There are 3 symptom nodes—“fatigue,” “cough,” and “lung”—represented by green nodes. These 3 nodes form a rule where the antecedents are “fatigue” and “cough,” and the consequent is “lung.” Both the nodes in the antecedent have outgoing links, which are pointed toward the R11 node. Similarly, there is an outgoing link in R11, which is pointing toward the consequent “lung” node.

**Table 7:** List of identified symptom rules.

Rule (R)	Antecedents	Consequents	Support	Confidence	Lift
R0	(loss_taste, lung)	(loss_smell)	0.0028	0.7748	14.5400
R1	(loss_taste, fatigue)	(loss_smell)	0.0028	0.7368	13.8281
R2	(loss_smell, lung)	(loss_taste)	0.0028	0.7107	15.3196
R3	(loss_smell, fatigue)	(loss_taste)	0.0028	0.6614	14.2564
R4	(loss_taste, brain_fog)	(loss_smell)	0.0018	0.5978	11.2192
R5	(loss_taste)	(loss_smell)	0.0272	0.5871	11.0173
R6	(loss_smell)	(loss_taste)	0.0272	0.5111	11.0173
R7	(loss_smell, brain_fog)	(loss_taste)	0.0018	0.4911	10.5847
R8	(heart, pain)	(lung)	0.0012	0.3684	2.3522
R9	(heart, brain_fog)	(lung)	0.0032	0.3542	2.2617
R10	(ache)	(fatigue)	0.0047	0.3471	1.9921
R11	(fatigue, cough)	(lung)	0.0012	0.3083	1.9686
R12	(heart, fatigue)	(lung)	0.0021	0.3014	1.9246
R13	(headache)	(fatigue)	0.0062	0.2675	1.5354
R14	(brain_fog, lung)	(heart)	0.0032	0.2574	2.6915
R15	(heart)	(lung)	0.0223	0.2328	1.4861
R16	(lung, pain)	(fatigue)	0.0016	0.2308	1.3245
R17	(insomnia)	(fatigue)	0.0013	0.2229	1.2791
R18	(muscle_pain)	(fatigue)	0.0019	0.2159	1.2392
R19	(muscle_pain)	(ache)	0.0019	0.2159	15.8929
R20	(cough)	(lung)	0.0061	0.2126	1.3572
R21	(lung, cough)	(fatigue)	0.0012	0.1989	1.1417
R22	(chest_pain)	(lung)	0.0053	0.1891	1.2075
R23	(fever)	(fatigue)	0.0046	0.1871	1.0737
R24	(pain)	(fatigue)	0.0102	0.1736	0.9962
R25	(cold)	(flu)	0.0064	0.1721	1.8483
R26	(lung, pain)	(heart)	0.0012	0.1683	1.7597
R27	(muscle_pain)	(lung)	0.0015	0.1667	1.0641
R28	(weak)	(fatigue)	0.0019	0.1633	0.9374
R29	(trauma)	(brain_fog)	0.0012	0.1586	0.6157
R30	(ache)	(lung)	0.0021	0.1553	0.9918
R31	(fatigue, pain)	(lung)	0.0016	0.1548	0.9886
R32	(ache)	(pain)	0.002	0.1505	2.5553
R33	(heart, lung)	(brain_fog)	0.0032	0.1422	0.5521
R34	(lung)	(heart)	0.0223	0.1421	1.4861
R35	(brain_fog, lung)	(fatigue)	0.0017	0.1421	0.8155
R36	(joint_pain)	(brain_fog)	0.0016	0.142	0.5514
R37	(ache)	(headache)	0.0019	0.1383	6.0025
R38	(ache)	(muscle_pain)	0.0019	0.1383	15.8929
R39	(cough)	(fatigue)	0.004	0.1371	0.7871
R40	(arm_pain)	(fatigue)	0.0021	0.135	0.7749
R41	(muscle_pain)	(pain)	0.0012	0.1326	2.2512
R42	(brain_fog, fatigue)	(lung)	0.0017	0.1274	0.8134
R43	(muscle_pain)	(weak)	0.0011	0.1212	10.533
R44	(weak)	(lung)	0.0014	0.1203	0.7684
R45	(fatigue, lung)	(heart)	0.0021	0.1198	1.2525
R46	(pain)	(lung)	0.0069	0.1165	0.7436
R47	(ache)	(fever)	0.0015	0.1141	4.6563
R48	(joint_pain)	(pain)	0.0013	0.113	1.9195
R49	(lung)	(fatigue)	0.0173	0.1107	0.6356
R50	(depress)	(brain_fog)	0.0081	0.1095	0.425
R51	(headache)	(lung)	0.0025	0.1087	0.6942
R52	(loss_smell, loss_taste)	(lung)	0.0028	0.1041	0.6647
R53	(depress)	(fatigue)	0.0076	0.1024	0.5877
R54	(loss_smell, loss_taste)	(fatigue)	0.0028	0.1017	0.5837
R55	(joint_pain)	(ache)	0.0012	0.1014	7.4676
R56	(fatigue, lung)	(brain_fog)	0.0017	0.1008	0.3912

## Discussion

### Principal Findings

The symptoms associated with LCS are still poorly understood. Analyzing social media conversations of patients related to long COVID allows us to understand the frequency and relationship between symptoms. Based on a large amount of Twitter social media data related to LCS, we performed the following 2 tasks in this paper. First, we identified the symptoms and medical conditions related to long COVID that were discussed on the Twitter social media platform. Second, we determined the patterns of symptoms and their associations.

Brain fog, fatigue, and breathing/lung issues were the 3 most common symptoms identified by the analysis. The literature sources verified these reported symptoms [3,4]. With a strong relationship, loss of taste and loss of smell were the most common consequent symptoms. We also identified a substantial number of meaningful symptom rules for a predefined threshold for confidence and support. Only the positively correlated relationships were considered by setting the lift to be greater than 1. The number of strong association rules can be changed according to different minimum thresholds. By finding associations among long COVID symptoms, this work will help physicians identify the behavior of the patients with long COVID. This paper provides new ideas for symptom mining and reveals the relationship between symptoms and their application value. Thus, the work has theoretical and practical importance.

We have used a novel data source, Twitter, and multiple NLP and machine learning techniques to explore the symptoms described by a large undifferentiated population. Different NLP methods, such as sentiment analysis, keyword extraction, and lemmatization, were used to extract information and symptoms from the unstructured text data. Subsequently, we used ARM concepts [20] to expose meaningful relationships between long COVID symptoms. This proof of concept has demonstrated the value of these techniques in describing the frequency of symptoms and the relationships between more common symptoms.

Future research can build on this methodology with clinical data sources such as electronic medical records, adding individual covariates such as sex, age, location, and comorbidities. This research can also be further extended to detect and predict the consequences of a given set of symptoms using word popularity detection methods.

### Limitations

This study is based on web-based Twitter data with limited patient-level variables. No information about the demographics of the tweet authors was available. Furthermore, we only considered patients who shared their experiences with the public in English, as filtering English-language tweets from multilingual tweets is computationally intensive. Another limitation of the study is that results can be affected by misinformation or false conversations on the Twitter platform.

There is a caveat for the confidence metric in the ARM technique when a negative correlation exists between the 2 sets, for instance, ∼ *X* → *Y*. In most cases, lower support and confidence are preferred when examining negatively correlated rules. The positive symptoms are often clear; however, negative symptoms are subtler and more difficult to recognize and diagnose. As a result, we have not investigated negatively correlated rules. Furthermore, rules discovered by algorithms require clinical validation and verification.

### Conclusion

The most frequent symptoms in our study included brain fog, fatigue, breathing/lung issues, heart issues, flu symptoms, and depression. General pains, loss of smell and taste, cold, cough, chest pain, fever, headache, and arm pain emerged in 1.6% to 5.3% of patients with long COVID. Furthermore, the highest confidence level–based detection with 10% minimum confidence and 0.01% minimum support successfully demonstrates the potential of association analysis and the Apriori algorithm to establish patterns to explore 57 meaningful relationship rules among long COVID symptoms. In this study, to identify the positively correlated symptoms, we only considered the rules with the lift being greater than 1. The results revealed that patients with lung/breathing problems and loss of taste are likely to have loss of smell with 77% confidence.

## Abbreviations

ARM: association rule mining
LCS: long COVID syndrome
NLP: natural language processing
NLTK: Natural Language Toolkit
PMI: Pointwise mutual information
